# Development and application of a novel model to predict the risk of non-alcoholic fatty liver disease among lean pre-diabetics with normal blood lipid levels

**DOI:** 10.1186/s12944-022-01752-5

**Published:** 2022-12-31

**Authors:** Wentao Zhu, Pei Shi, Jiwei Fu, An Liang, Ting Zheng, Xiaoping Wu, Songsong Yuan

**Affiliations:** Department of Infectious Diseases, the First Affiliated Hospital of Nanchang University, No. 17 Yongwai Street, Donghu District, Nanchang, China

**Keywords:** Non-alcoholic fatty liver disease, Pre-diabetes, Screening tool, Nomogram, Least absolute shrinkage and selection operator regression analysis

## Abstract

**Background:**

Non-alcoholic fatty liver disease (NAFLD) has been associated with type 2 diabetes, but its relationship with pre-diabetes is still unknown. This study aims to determine whether pre-diabetes is associated with NAFLD, followed by establishing a NAFLD predictive nomogram for lean Chinese pre-diabetics with normal blood lipids.

**Methods:**

Datasets from 3 previous studies, 1 (2774 pre-diabetics with normal blood lipids for training, 925 for validation), 2 (546 for longitudinal internal validation, post-5-year follow-up), and 3 (501 from another institution for external validation), were used. Kaplan-Meier determined cumulative NAFLD hazard, and least absolute shrinkage and selection operator regression analysis uncovered its risk factors. Multivariate logistic regression analysis constructed the nomogram, followed by validation with receiver operating characteristic curve, calibration plot, and decision curve analyses.

**Results:**

NAFLD incidence increased with diabetes progression, and pre-diabetics had higher cumulative risk versus non-diabetics, even for lean individuals with normal blood lipids. Six risk factors were identified: body mass index, total cholesterol, alanine aminotransferase:aspartate aminotransferase, triglyceride:high density lipoprotein cholesterol, fasting blood glucose and γ-glutamyl-transferase. The nomogram yielded areas under the curve of 0.808, 0.785, 0.796 and 0.832, for respectively, training, validation, longitudinal internal validation, and external validation, which, along with calibration curve values of *p* = 0.794, 0.875, 0.854 and 0.810 for those 4 datasets and decision curve analyses, validated its clinical utility.

**Conclusions:**

Lean pre-diabetic Chinese with normal blood lipids have higher NAFLD risk versus non-diabetics. The nomogram is able to predict NAFLD among such individuals, with high discrimination, enabling its use for early detection and intervention.

**Supplementary Information:**

The online version contains supplementary material available at 10.1186/s12944-022-01752-5.

## Introduction

Non-alcoholic fatty liver disease (NAFLD) is characterized by excessive fat accumulation within the liver not stemming from known causes, such as heavy alcohol consumption and viral hepatitis. Its severity ranges from simple steatosis (no significant liver inflammation/hepatocyte damage) to advanced cirrhosis and hepatocellular carcinoma [1–4]. NAFLD is considered a liver manifestation of metabolic syndrome, often associated with type 2 diabetes mellitus (T2DM) and obesity [5, 6]. Indeed, increasing evidence has found NAFLD being an independent risk factor for T2DM, which itself could contribute to worsening NAFLD in a vicious cycle. This is further supported by NAFLD prevalence among T2DM individuals being 40–70%, significantly higher than for the overall global population at 25% [7–11].

Recent studies, however, have shown that NAFLD and T2DM are equally common in lean (body mass index [BMI] < 23 kg/m^2^) Asians, despite the long-standing association of obesity with these disorders, possibly owing to lifestyle, gut microbiota, genetic, and environmental factors [12–15]. Epidemiological studies have indicated that ~ 10–20% of NAFLD individuals were lean [16]; these individuals are at increased risk for T2DM onset and mortality, compared to obese NAFLD individuals [17, 18]. Yet, because of NAFLD often being associated with obesity, NAFLD in lean patients, especially pre-diabetics with normal blood lipid levels, are often overlooked until liver damage has progressed to the point of developing symptoms. Currently, liver biopsy is the gold standard for NAFLD diagnosis, but it has significant limitations due to high expenses, invasiveness, as well as risks for sampling errors and complications [19]. Therefore, a non-invasive approach for diagnosing and/or predicting NAFLD onset has been of great interest; numerous researchers have developed various predictive models, using a variety of biomarkers, such as from Zhang et al., whose model incorporated gender, age, BMI, triglycerides (TG) and other indicators to evaluate NAFLD risk in T2DM [20]. However, few studies have been conducted regarding the relationship between prediabetes and NAFLD, particularly NAFLD risk among lean pre-diabetics with normal blood lipid levels.

This study aims to fill in this gap by examining the associations between lean pre-diabetic Chinese individuals with normal blood lipid levels and NAFLD onset. Factors linked to increased NAFLD risk were elucidated, and a new predictive nomogram model was developed and verified, particularly with respect to clinical settings. The nomogram predicted the likelihood of NAFLD onset among lean pre-diabetics with normal blood lipid levels, and was highly capable of discriminating between those who were and were not at risk for developing NAFLD, indicating its usefulness as a non-invasive approach for NAFLD screening and facilitation of early interventional strategies.

## Methods

### Patient data and variable measurements

All data are freely available from the “DATADRYAD” database; the authors [21, 22] who initially collected this data have transferred ownership to the database owner, granting us permission to use this data for secondary analysis. Measurement data were obtained for the following variables: gender, age, BMI, NAFLD incidence, height, waist circumference (WC), diastolic (DBP) and systolic blood pressures (SBP), γ-glutamyl-transferase (GGT), alanine (ALT) and aspartate (AST) aminotransferase, ALT:AST ratio (AAR), total protein (TP), globulin (GLB), albumin (ALB), fasting blood glucose (FPG), total cholesterol (TC), TG, high (HDL-C) and low-density lipoprotein cholesterol (LDL-C), TG:HDL-C ratio (THR), total (TB) and direct bilirubin (DBIL), alkaline phosphatase (ALP), hemoglobin A1c (HbA1c), creatinine (Cr), uric acid (UA), blood urea nitrogen (BUN), as well as smoking status, being a regular exerciser, and duration of the follow-up period.

### Study design and populations

**Fig. 1:**
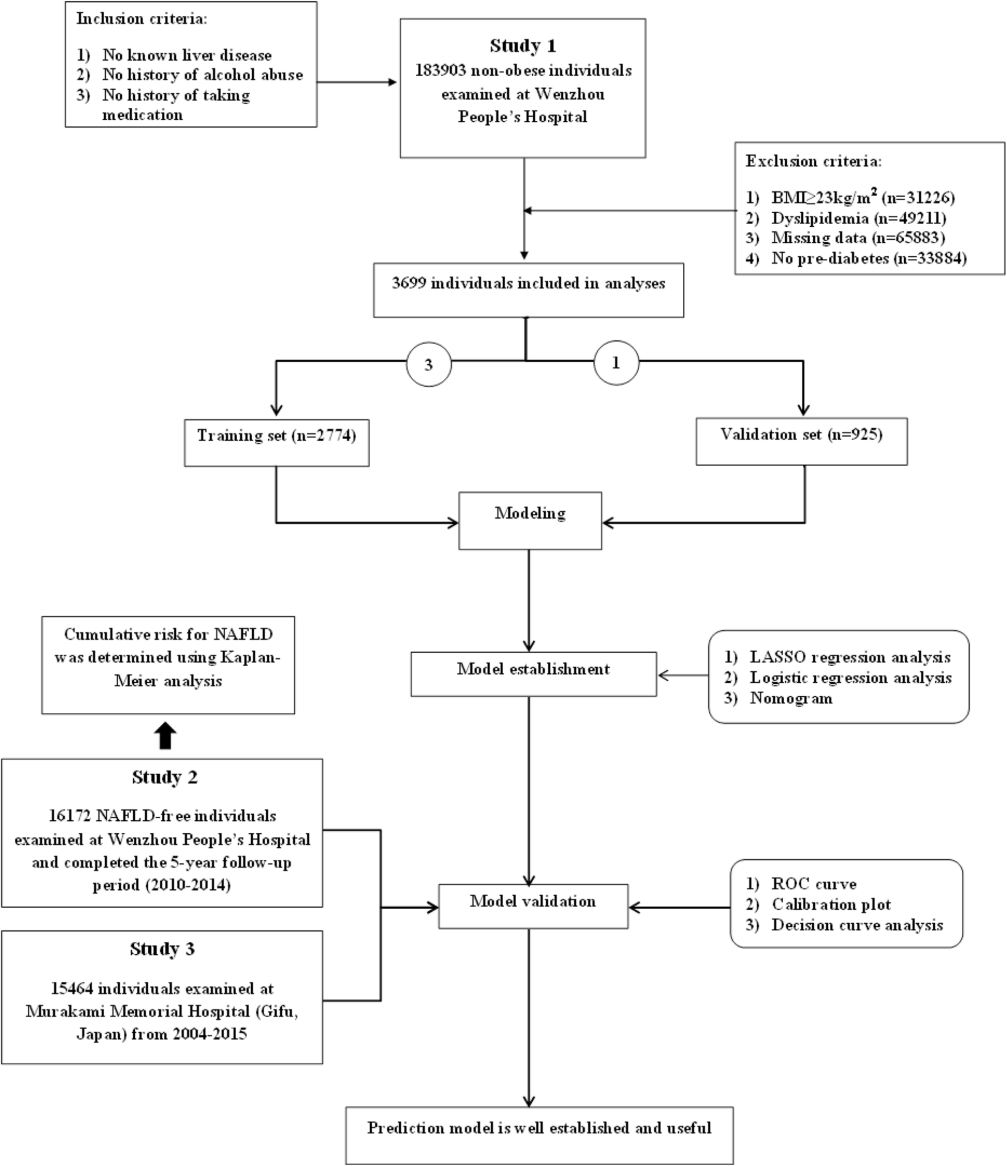
Flow chart of the study design to establish the predictive nomogram for non-alcoholic fatty liver disease (NAFLD) onset among pre-diabetic non-obese Chinese individuals with normal blood lipid levels

This study consists of a secondary analysis of 3 longitudinal or cross-sectional studies, of which Studies 1 and 2 were conducted in Wenzhou People’s Hospital. Study 1 was a cross-sectional study, enrolling 183,903 non-obese individuals, while Study 2 was a longitudinal one enrolling 16,172 non-obese individuals, initially NAFLD-free, who completed a 5-year follow-up examination. By contrast, Study 3 enrolled 15,464 Japanese individuals, based on the NAGALA (NAFLD in the Gifu Area, Longitudinal Analysis) database, previously used to investigate the effect of obesity on T2DM risk. For all 3 studies, subjects fulfilling the following baseline inclusion criteria were included: 1) No known liver disease, 2) No alcohol abuse (< 40 g/day or < 70 g/week for females, < 60 g/day or < 140 g/week for males), 3) No medication history. Exclusion criteria were also applied: 1) Dyslipidemia (TC > 5.2 mmol/L, TG > 1.7 mmol/L, LDL-C > 3.12 mmol/L, HDL-C < 1.03 mmol/L), 2) BMI ≥ 23 kg/m^2^, and 3) Missing subject data. After applying inclusion and exclusion criteria, 37,581 subjects were included in Study 1 (3699 pre-diabetic, based on FPG = 5.84 [5.69–6.10]), 7897 in Study 2 (642 pre-diabetic, of which 546 with GGT data available were incorporated as part of the internal longitudinal validation set, with FPG = 5.85 [5.70–6.11]) and 4908 in Study 3 (501 pre-diabetic, based on FPG = 5.66 [5.55–5.83] or HbA1c around 5.70%; Fig. 1).

### Diagnosis of NAFLD and pre-diabetes

Subjects were evaluated annually by liver ultrasound for NAFLD, based on criteria outlined by the Chinese Liver Disease Association in 2010, [23] entailing diffuse hyper-echogenicity of the liver, compared to spleen and kidney, combined with one of the following characteristics: 1) Unclear intrahepatic structure, 2) Enlarged liver with a round, blunt border, 3) Unclear/incomplete right liver lobe and diaphragm, or 4) Weakened hepatic blood flow signal, but with normal blood flow distribution. Pre-diabetic subjects were defined as having either FPG 5.6–6.9 mmol/L or HbA1c 5.7–6.4%.

### Constructing the predictive nomogram and statistical analysis

The results were reported in line with Transparent Reporting of a Multivariable Prediction Model for Individual Prognosis or Diagnosis (TRIPOD) [24]. For establishing and verifying the predictive nomogram, 3669 pre-diabetic patients in Study 1 were randomly divided into 2 cohorts: 2774 in the development cohort, and 925 in the validation cohort, in line with the optimal theoretical ratio of 3:1, to construct the nomogram. For verification, 546 subjects from Study 2, who completed the 5-year follow-up period, were included as part of the internal longitudinal validation set, while the external validation set consisted of 501 subjects from Study 3.

When comparing baseline characteristics for each group, continuous variables were displayed as mean ± SD, and categorical variables as median (quartile). Significant differences between groups were determined, using either a non-parametric one-way ANOVA test for continuous variables, or χ2 test for categorical variables. *P* < 0.05 was considered statistically significant. Kaplan-Meier analysis was used to calculate the cumulative hazard for NAFLD over time, in order to evaluate the relationship between diabetes progression and NAFLD; this was conducted by the survival package of R software (version 4.1.3 3). To obtain the predictors behind NAFLD, least absolute shrinkage and selection operator (LASSO) regression analysis was run by glmmet package, which were then subjected to multivariate logistic regression analysis by rms package to build the predictive nomogram. The statistical values for those predictors were expressed in terms of odds ratios (OR), with a 95% confidence interval (CI). This nomogram was then evaluated using receiver operator characteristic curve (ROC) analysis, using pROC package, to determine its discriminatory capability, in which the closer the area under the ROC curve (AUC) was to 1, the greater the accuracy of the nomogram. Calibration curves were then constructed by rms to determine the extent of agreement between predicted probabilities and actual observations for NAFLD. Clinical utility of the nomogram was determined using decision curve analysis (DCA), in which standardized net benefits were calculated under different threshold probabilities.

## Results

### Characteristics of the cohort subjects

The baseline characteristics for Study 1 are summarized in Supplementary Table S1, in which 37,581 lean individuals (16,053 males, 21,528 females), with normal blood lipid levels, were included in the total cross-sectional cohort. Median age was 36 years (IQR 30–47) and 1364 had NAFLD (3.6%). The cohort was then divided into 3 groups, based on standard criteria for diabetes: 33209 normal, 3699 pre-diabetics, and 673 diabetics. NAFLD was highest in both diabetic (14.9%) and pre-diabetics (7.8%), compared to normal (2.9%). Furthermore, compared to normal, pre-diabetics were older, had higher BMI and less favorable biochemical marker levels. In fact, the differences in clinical parameters between the 3 groups were all statistically significant (*P* < 0.001; Table S1). The pre-diabetic group, in turn, was then randomly divided into 2 cohorts: 2774 in development, and 925 in validation cohorts. No statistically significant differences were found between these 2 cohorts in terms of baseline demographics, clinical characteristics and NAFLD incidence (Table S2).

**Fig. 2:**
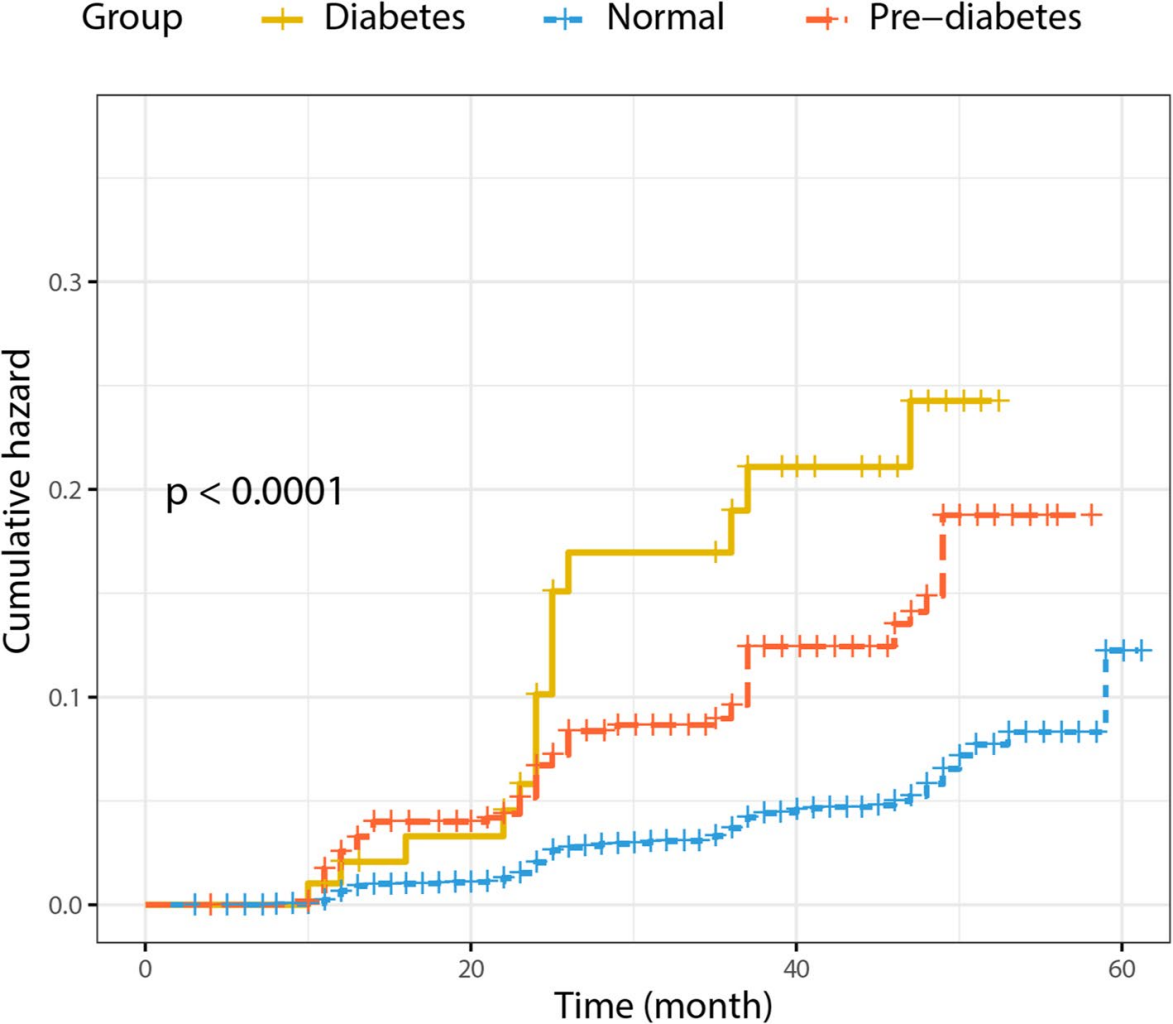
Kaplan-Meier analysis of the cumulative risk for developing NAFLD among non-diabetics (blue), pre-diabetics (red), and diabetics (yellow) over the 5-year follow-up period. Diabetes progression was found to be positively associated with increased risk of NAFLD onset. *p* < 0.0001 between all 3 groups

Supplementary Table S3 displays baseline characteristics for Study 2, comprising 7897 initially NAFLD-free individuals, who attended annual health check-ups at Wenzhou People’s Hospital during a 5-year follow-up period. At the end of that period, 343 were diagnosed with NAFLD. All subjects were divided into normal, pre-diabetic and diabetic groups, in which NAFLD increased from 1 group to the next, with 3.7% among non-diabetics, 9.7% in pre-diabetics, and 15.3% in diabetics. Diabetes was thus found to be positively correlated with increased NAFLD risk under Kaplan-Meier analysis; in particular, pre-diabetics had higher cumulative risk throughout the 5-year follow-up period for NAFLD, compared to normal, while diabetics had the highest risk (Fig. 2). An internal validation set was then established, using 546 pre-diabetics, and it was found that 10.4% of them had NAFLD.

In Study 3, 4908 individuals examined at Murakami Memorial Hospital (Gifu, Japan) from 2004 to 2015 were included, of which 143 had NAFLD, and the remaining 4765 did not (Table S4). Median age was 43 years (IQR 39–50), which was older than the 39 years of non-NAFLD patients (IQR 35–45; *P* < 0.001). Additionally, the follow-up period for NAFLD patients was longer than for non-NAFLD group (2147 vs 2037 days, *P* < 0.001). Afterwards, 501 pre-diabetics were included in the external validation set, in which 39 had NAFLD, and median follow-up was 1638 days (IQR 734.5–2926).

### Development of the predictive nomogram

**Fig. 3:**
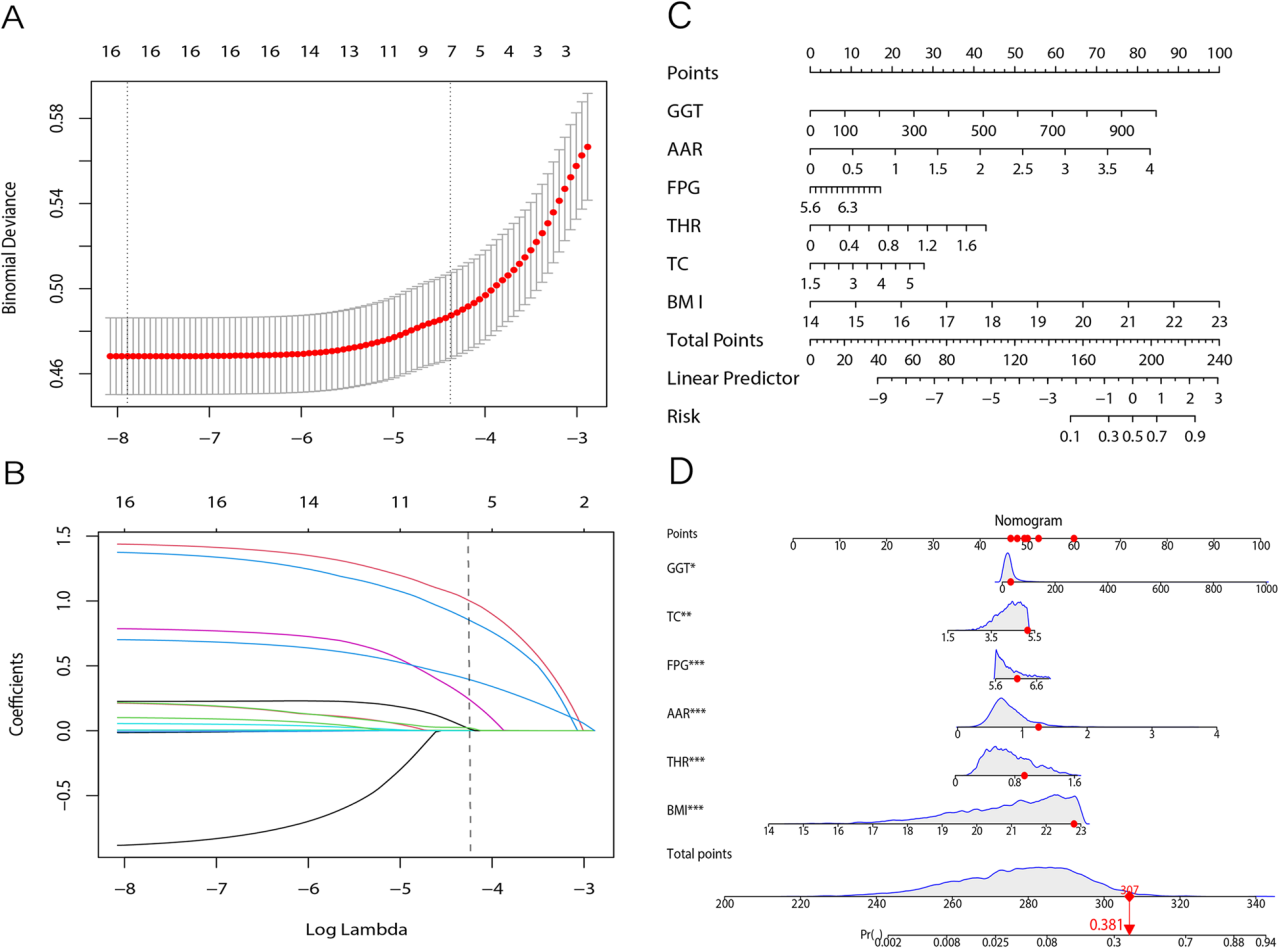
Selection of variables using the least absolute shrinkage and selection operator (LASSO) binary logistic regression model. A coefficient profile plot was constructed against the log (lambda) sequence. **A** Seventeen variables with nonzero coefficients were selected by deriving the optimal lambda value. **B** Following verification of the optimal parameter (lambda) in the LASSO model, partial likelihood deviance (binomial deviance) curve versus log (lambda) was plotted, and dotted vertical lines for those variables were drawn, based on 1 standard error criteria, to obtain the 7 variables (body mass index [BMI], total cholesterol [TC], alanine aminotransferase to aspartate aminotransferase ratio [AAR], triglyceride to high density lipoprotein cholesterol ratio [THR], fasting blood glucose [FPG], γ-glutamyl-transferase [GGT], and uric acid [UA]). Construction of the predictive nomogram. **C** The predictive nomogram is based on the risk factors of GGT, AAR, FPG, BMI, THR, TC, and BMI. **D** Example of a nomogram in use, where the patient measurements for each of the 6 parameters corresponds to a specific point value, and the total points corresponds to a percentage likelihood of developing NAFLD

Initially, 17 variables were included at the start of the construction of the predictive nomogram: gender, age, BMI, GGT, AAR, TP, GLB, ALB, FPG, TC, THR, LDL-C, TB, DBIL, Cr, UA, and BUN. LASSO regression analysis reduced the number of potential variables for the nomogram from 17 to 7, comprising BMI, TC, AAR, THR, FPG, UA and GGT. Figure 3A and B shows the correlations between regression coefficients and LASSO’s lambda. These 7 variables were then incorporated into logistics regression analysis, whose results are displayed in Table 1, and out of those 7, only UA was excluded from the final predictive nomogram. The remaining 6 variables were then used as the basis for the predictive nomogram in Fig. 3C. The operation of this nomogram is through each of those 6 variables corresponding to a specific points value, and the total points added from those variables, in turn, corresponds to a specific probability for developing NAFLD. For example, a non-obese pre-diabetic with normal blood lipid levels, possessing a BMI of 22.801 kg/m^2,^ GGT of 32 U/L, AAR of 1.25, FPG of 6.13 mmol/L, TC of 5.17 mmol/L, and THR of 0.929, has an estimated probability of NAFLD of 38.1% (Fig. 3D).

**Table 1:** Multivariate logistic regression analysis of NAFLD risk predictors among non-obese pre-diabetics with normal blood lipid levels

Variables	Estimate	Standard error	*z*	*P*	Odds ratio	Confidence Interval (2.5%)	Confidence Interval (2.5%)
Intercept	−25.958	2.326	−11.162	< 0.001	0.000	0.000	0.000
GGT	0.004	0.002	2.121	0.034	1.004	1.001	1.009
ALT/AST	1.267	0.222	5.702	< 0.001	3.551	2.293	5.490
FPG	0.797	0.213	3.740	< 0.001	2.218	1.455	3.357
TG/HD-C	1.359	0.250	5.442	< 0.001	3.893	2.387	6.357
TC	0.422	0.138	3.054	0.002	1.525	1.168	2.009
UA	0.001	0.001	1.565	0.117	1.001	9.996	1.003
BMI	0.656	0.081	8.125	< 0.001	1.928	1.654	2.271

*ALT* alanine aminotransferase, *AST* aspartate aminotransferase, *BMI* body mass index, *FPG* fasting plasma glucose, *GGT* γ-glutamyltranspeptidase, *HDL-C* high-density lipoprotein cholesterol, *NAFLD* nonalcoholic fatty liver disease, *TC* total cholesterol, *TG* triglyceride, *UA* uric acid

### Evaluating discriminatory capability and accuracy for the predictive nomogram

**Fig. 4:**
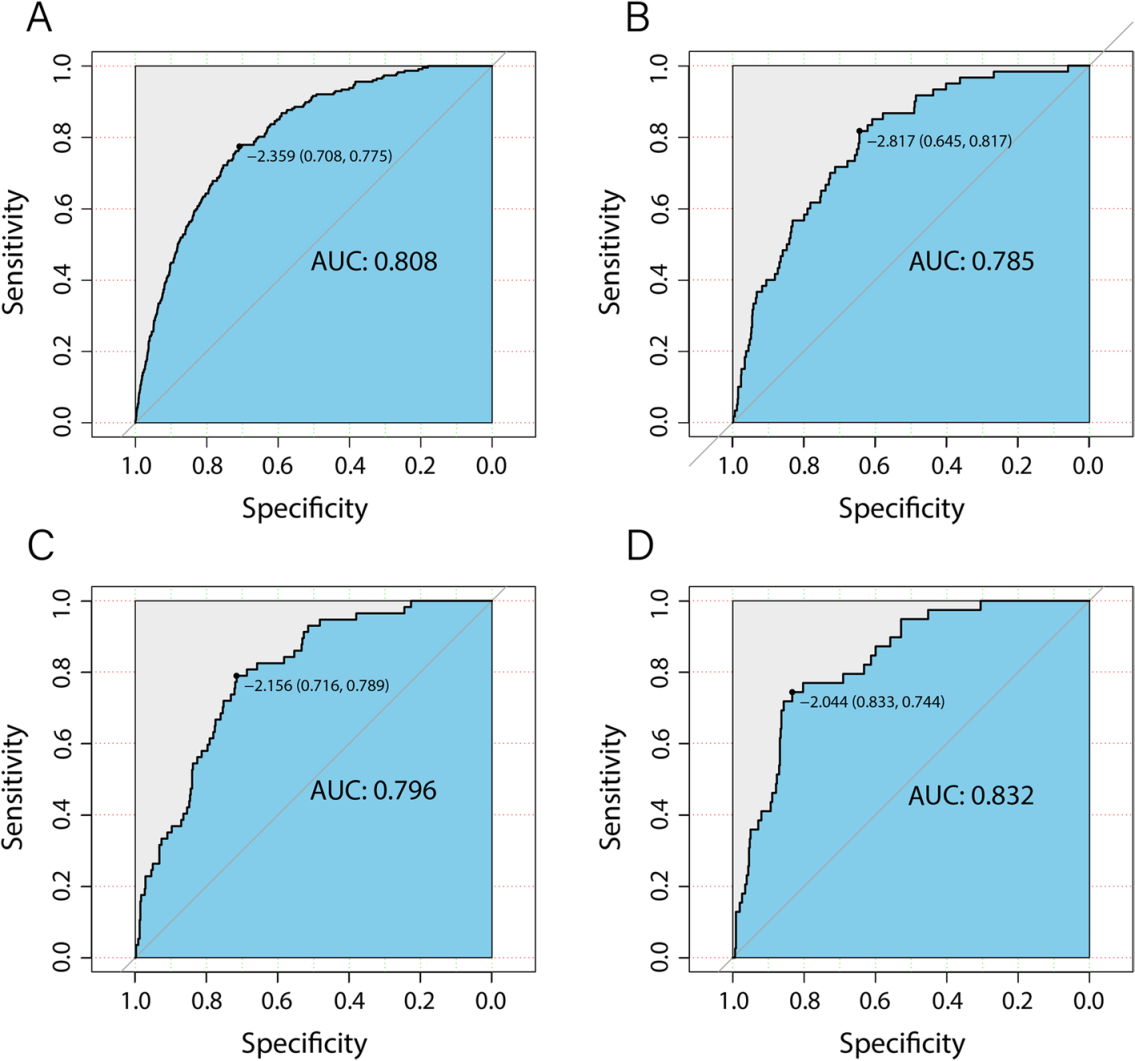
Validation of the accuracy of the predictive nomogram using receiver operating characteristic curve (ROC) analysis, based on data from individuals in **A** training, **B** validation (both Study 1), **C** longitudinal internal validation (Study 2), and **D** external validation (Study 3) sets

**Fig. 5:**
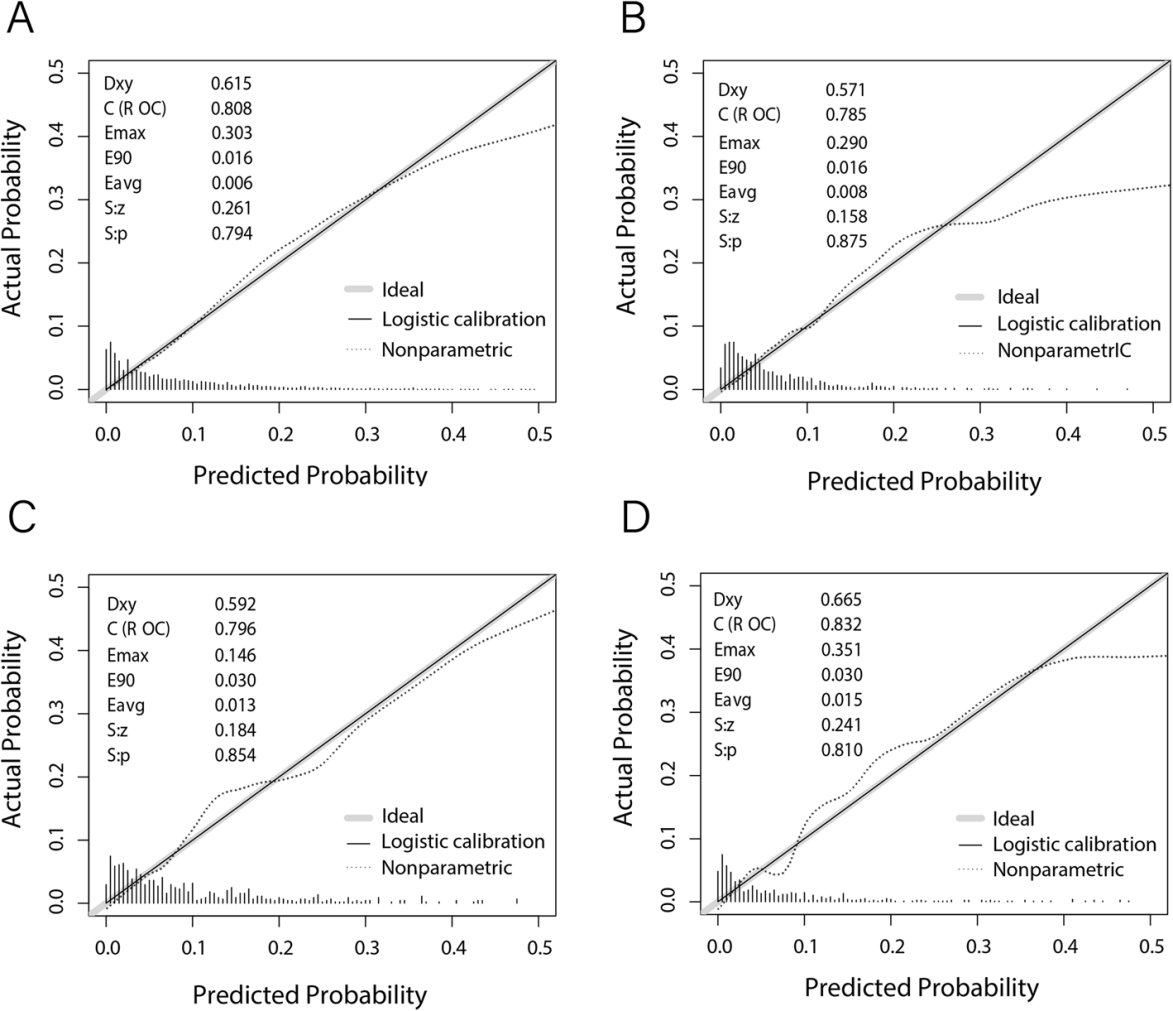
Calibration curves demonstrating the correspondence between the predicted outcomes from the nomogram, compared to the actual results in **A** training, **B** validation (both Study 1), **C** longitudinal internal validation (Study 2), and **D** external validation (Study 3) sets

**Fig. 6:**
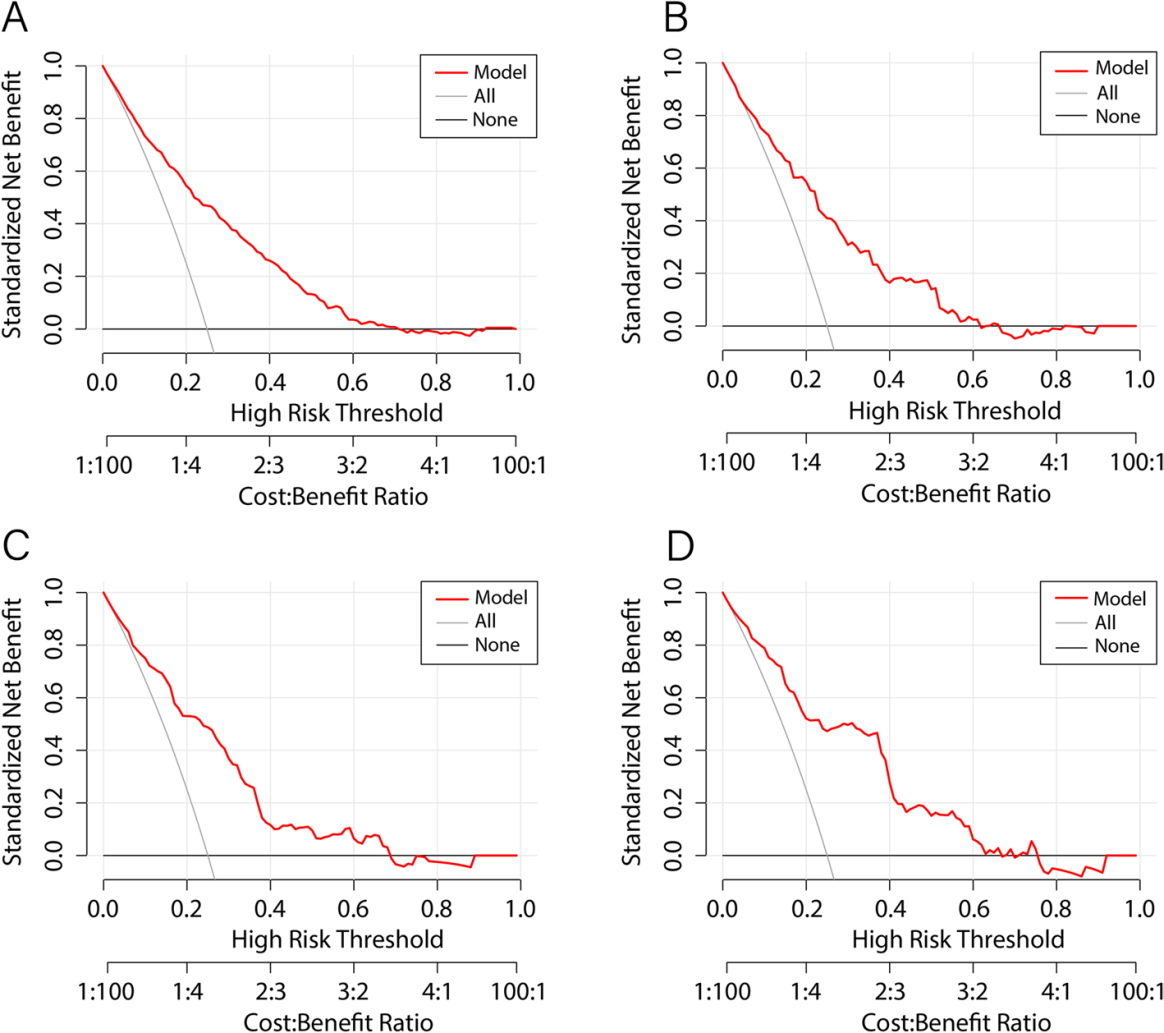
Decision curve analysis of the predictive nomogram, based on data from the **A** training, **B** validation (both Study 1), **C** longitudinal internal validation (Study 2), and **D** external validation (Study 3) sets. Red line represents the predictive nomogram, thin solid line the hypothesis that all patients are diagnosed with NAFLD, and thick solid line the hypothesis that no patients are diagnosed with NAFLD

ROC curves were used to determine the discriminatory capability and accuracy of the predictive nomogram, and the resulting AUCs were shown in Fig. 4, for the training (Fig. 4A), validation (Fig. 4B), longitudinal internal validation (Fig. 4C), and external validation sets (Fig. 4D). All of these results, yielding AUCs of 0.785–0.832, indicated that the nomogram was highly discriminatory for detecting NAFLD occurrence. Calibration curves were then used to evaluate the correspondence between the predicted values from the nomogram with the actual probability for developing NAFLD (Fig. 5). The correspondence between predicted and actual probabilities for the training, validation, longitudinal internal validation, and external validation were, respectively, *P* = 0.794 (Fig. 5A), 0.875 (Fig. 5B), 0.854 (Fig. 5C), and 0.810 (Fig. 5D), indicating that the results from the nomogram had a high degree of concurrence with the actual findings, as no significant difference was found between predicted and actual probabilities. DCA showed that the threshold probability of the model in the training (Fig. 6A), validation (Fig. 6B), longitudinal internal validation (Fig. 6C) and external validation sets (Fig. 6D) were all higher than for “all patients with NAFLD” or “no patients with NAFLD”, indicating that the nomogram was of clinical utility. For instance, in Fig. 6A, a NAFLD risk probability of 40% corresponded to a net benefit of ~ 25%, which could be interpreted that the nomogram would benefit ~ 25 out of 100 individuals.

## Discussion

Obesity and dyslipidemia used to be considered the main risk factors for NAFLD, as well as increasing T2DM and cardiovascular disease risk in NAFLD [25]. However, owing to lean individuals, particularly from Asian populations, being diagnosed with NAFLD [15, 26], the concept of lean NAFLD has received increasing attention. In fact, surprisingly, lean individuals with NAFLD are at greater risk for T2DM, and have higher mortality rates, compared to obese individuals [17, 18]. The association between T2DM and NAFLD is not surprising, as NAFLD has been found to be an independent risk factor for T2DM, playing a significant role in its development; indeed, a meta-analysis from Ballestri et al. found that NAFLD was associated with an almost 2-fold increased risk for T2DM during a 5-year follow-up period [27]. There is also a reciprocal relationship between T2DM presence and NAFLD risk, in which NAFLD is more prevalent among T2DM individuals, compared to the overall global prevalence of NAFLD [10]. However, while the link between T2DM and NAFLD has been well-established, the association between NAFLD and pre-diabetes is poorly-defined. In this study, it was demonstrated that among lean individuals with normal blood lipid levels, prediabetics were more at risk for NAFLD, compared to normal individuals, as shown in the results from Study 2, where 7897 initially NAFLD-free, lean Chinese individuals with normal blood lipid levels were divided into normal, pre-diabetic, and diabetic groups. There, NAFLD incidence at the end of the 5-year follow-up period increased from as low as 3.7% among normal, to 9.7% in pre-diabetics and 15.3% in diabetics, suggesting that the latter 2 categories were more likely to develop NAFLD, compared to normal. This was further supported by Kaplan-Meier analysis demonstrating a positive correlation between diabetes progression and increased NAFLD risk; indeed, pre-diabetics had higher cumulative NAFLD risk after the 5-year follow-up period, compared to normal. This increased risk for NAFLD among pre-diabetic lean Chinese individuals, with normal blood lipid levels, was associated with 6 risk factors: BMI, TC, AAR, THR, FPG and GGT, which were then incorporated as part of a predictive nomogram, yielding an AUC ~ 0.8 among the 4 sets tested, indicating that it had a high discriminatory capability; this nomogram was further validated by calibration curves. The clinical utility of the nomogram was confirmed by DCA, indicating that it could be used for screening of high-risk individuals, allowing earlier and more effective interventions against NAFLD.

“Pre-diabetes” describes a condition where blood glucose levels are higher than normal, but lower than those associated with T2DM diagnosis. The pathogenesis underlying this condition stems from impaired β-cell function and increased insulin resistance (IR). Prediabetic cases have been increasing worldwide in an alarming trend [28], and is even more prevalent among Asians, compared to Westerners [29]. As a result, prediabetes is regarded as a critical stage, as early screening and intervention could reduce, or even reverse, the risk of progressing to diabetes. Such screening and intervention during prediabetes could also potentially aid in reducing NAFLD risk [30, 31]; however, the prediabetic population, particularly those who are lean, are often ignored in clinical practice until they had already progressed to T2DM or NAFLD.

To meet this unmet need, we thus developed a novel predictive nomogram to determine NAFLD risk among pre-diabetic lean Chinese individuals with normal blood lipid levels. There have already been several predictive models developed to determine the risk of individuals ending up with T2DM and NAFLD, such as from Zhang et al. and Xue et al. [20, 32], both of which focused on assessing NAFLD risk among Chinese T2DM. Another model from Cai et al. [33] is able to estimate 8-year incidence of T2DM among NAFLD populations. However, these models did not deal with predicting NAFLD risk among pre-diabetics, unlike our nomogram. In this study, BMI, TC, AAR, THR, FPG and GGT were used as the basis for the nomogram, as they were considered the most predictive under LASSO and logistic regression analyses. Both LASSO and logistic regression are able to solve all kinds of problems involving multicollinearity and confounding factors, providing more accurate results compared to other analytical methods. Additionally, compared with the traditional prediction model, the nomogram model is more accurate, easier to visualize, and more convenient for clinical decision-making. This was then verified by establishing 4 data sets: training, validation, longitudinal internal validation, and external validation. Furthermore, ROC and calibration curve results, as well as DCA, confirmed that our nomogram was highly accurate with respect to its predictions when compared to actual outcomes, as well as providing greater utility in clinical settings for prognosticating future NAFLD. It has been noted, though, that current non-invasive diagnostic techniques, such as ultrasonic liver imaging and measurement of serum biomarkers, have already been proven to be useful for diagnosing NAFLD. However, both of these methods have limitations, in that “gold standard” cut-off values for serum biomarkers, such as AAR, have not been fully defined and validated. Furthermore, these biomarkers are not liver-specific, meaning that they could be influenced by co-morbidities, resulting in misleading measurements [34]. Additionally, ultrasound detection is less effective in the extremely early stages of NAFLD, limiting its utility for facilitating early intervention against this disease [35]. By contrast, the predictive nomogram established in this study was able to predict NAFLD onset long before its occurrence, even before it was detectable by either serum biomarkers or ultrasound.

### Comparisons with other studies and what the current work add to existing knowledge

The inclusion of the 6 parameters, BMI, TC, THR, AAR, FPG and GGT, in the predictive nomogram was in line with findings from previous studies. Obesity has been considered a significant independent risk factor for T2DM and NAFLD [36–38]; moreover, even in non-obese individuals, defined as having BMI < 25 kg/m^2^, increases in NAFLD risk has been found to be positively associated with BMI increases [39]. This is consistent with what was found in this study among lean Chinese individuals, whose BMI were < 23 kg/m^2^. Lipid-based metabolic disorders and adipose tissue dysfunction also play important roles in NAFLD onset, and close associations have been found between NAFLD occurrence and increased levels of TC, TG, HDL-C, and other lipid components [24, 40]. In particular, THR has been shown to be independently associated with NAFLD onset in healthy individuals, which is in line with it being a surrogate indicator of IR, and thus the progression of an individual towards prediabetic and diabetic stages, as well as NAFLD. This association, in turn, enables THR to serve as a NAFLD predictor, in which the higher the ratio, the higher the risk for developing NAFLD and diabetes [41, 42]. Both lipid-associated parameters and THR correlating to NAFLD risk was supported by this study, which demonstrated that higher NAFLD risk was present among those with higher TC and THR, even if they otherwise had overall normal lipid levels. As for GGT, ALT, and AST, they have long been used in China as liver functional indicators to evaluate hepatobiliary diseases. GGT is found on the surface of multiple cell types and is highly active in the liver, where it is involved in reducing oxidative stress. It is believed to be closely related to liver steatosis and fat deposition, and could possibly serve as a surrogate marker for NAFLD [43]. Additionally, epidemiological studies have confirmed that serum GGT is closely related to T2DM, possibly serving as an important predictive risk indicator [44]. ALT and AST are both specific markers of liver inflammation and cell damage, and are also closely related to NAFLD, likely owing to higher ALT and AST contributing to chronic liver inflammation, IR, and hepatic steatosis [45]. Compared to ALT and AST alone, though, AAR is more strongly predictive for NAFLD onset, which has led to its increased prevalence as a predictive indicator [46]; this was further supported by a study of 12,127 initially non-obese, NAFLD-free individuals, where AAR was found to be an independent risk factor for NAFLD onset in obese individuals [47]. FPG levels have also been found to reflect the level of secretion and functioning of basal insulin, leading to it being considered an independent predictor of DM [48, 49]. The current study has extended this observation to NAFLD, in that higher FPG has been found to correspond to greater NAFLD risk.

Furthermore, this study demonstrated that among lean Chinese individuals with normal blood lipid levels, prediabetics were more at risk for NAFLD, compared to normal individuals. This higher risk was able to be predicted, with high discriminatory capability, using a nomogram incorporating 6 factors: BMI, TC, AAR, THR, FPG and GGT. This nomogram could be thus used as a screening tool for identifying high-risk individuals, allowing earlier and more effective interventions against NAFLD.

### Study strengths and limitations

To the best of our knowledge, the present study is the first to develop and evaluate a predictive nomogram for NAFLD risk, among a lean Chinese population with normal lipid levels in the pre-diabetic stages. This nomogram was based on, and confirmed by, representative large sample populations obtained from different medical institutions in different regions of China and Japan, demonstrating its validity for a variety of different population groups. It was also based on findings from both cross-sectional and longitudinal studies, providing greater reliability in predicting NAFLD in a long-term time scale. Furthermore, the measurements for the 6 parameters in the predictive nomogram can be obtained simply and non-invasively, facilitating widespread ease in its adoption in clinical practice. However, there are a number of limitations to this study, one of which is that this was a secondary retrospective analysis, based on data collected from 3 previous studies, resulting in limitations in the data collected despite the large sample sizes. These limitations in the collected data included the number of times that FPG and HbA1c measurements were taken among the patients in those studies, as different conditions could affect FPG and HbA1c measurements, and thus patient categorization as normal, pre-diabetic, or diabetic. Additionally, NAFLD staging data was not available, even though it had been previously documented that a number of factors could have varying impacts at different NAFLD stages. For instance, it has been documented that the negative impact of dyslipidemia is less significant in later stages of NAFLD, when cirrhosis develops, due to the failure of hepatic lipid-synthesizing mechanisms at that stage [50]. Another limitation is that diabetes and pre-diabetes diagnostic criteria was mainly based on FPG, which could lead to undercounting, as FPG may miss some individuals who could otherwise be caught by other tests, such as the oral glucose tolerance test (OGTT), which was not carried out by the studies included in this paper. Future investigations should take OGTT, as well as multiple FPG and HbA1c measurements under multiple different conditions, to ensure that the overall values are fully reflective of patient glycemic statuses. Furthermore, the associations between the 6 factors incorporated into the predictive nomogram with different stages of NAFLD should be examined.

## Conclusions

NAFLD risk is higher in lean Chinese prediabetics with normal blood lipid levels, compared to normal individuals. A predictive nomogram was developed, incorporating the 6 strongest predictive parameters of BMI, TC, AAR, THR, FPG, UA and GGT, that was highly discriminatory between pre-diabetics who would develop NAFLD versus those who would not. This approach will facilitate the development of early screening and interventional strategies against NAFLD onset in pre-diabetics, ensuring greater preservation of liver functioning and better quality of life.

## Supplementary Information

**Additional file 1: Table S1**. Baseline characteristics of the participants in the cross-sectional Study 1 (*N* = 37,581). **Table S2**. Characteristics of the 3699 pre-diabetic individuals from Study 1, divided into training and validation sets. **Table S3**. Characteristics of the 7897 individuals enrolled in longitudinal Study 2, and 546 patients in the internal validation set. **Table S4**. Characteristics of the 4908 patients enrolled in external longitudinal cohort study 3, and 501 patients enrolled in the external validation set.

## Data Availability

All data generated or analyzed during this study are included in this published article (and its supplementary information files), as well as being available from the corresponding author on reasonable request.

## Abbreviations

AAR: ALT to AST ratio
ALB: Albumin
ALP: Alkaline phosphatase
ALT: Alanine aminotransferase
AST: Aspartate aminotransferase
AUC: Area under the curve
BMI: Body mass index
BUN: Blood urea nitrogen
CI: Confidence interval
Cr: Creatinine
DBIL: Direct bilirubin
DBP: Diastolic blood pressure
DCA: Decision curve analysis
FPG: Fasting plasma glucose
GGT: γ-glutamyltranspeptidase
GLB: Globulin
HbA1c: Hemoglobin A1c
HDL-C: High-density lipoprotein cholesterol
LASSO: Least absolute shrinkage and selection operator
LDL-C: Low-density lipoprotein cholesterol
NAFLD: Nonalcoholic fatty liver disease
OR: Odds ratio
ROC: Receiver operator characteristic
SBP: Systolic blood pressure
T2DM: Type 2 diabetes mellitus
TB: Total bilirubin
TC: Total cholesterol
TG: Triglyceride
THR: TG to HDL-C ratio
TP: Total protein
UA: Uric acid
WC: Waist circumference
OGTT: Oral glucose tolerance test
