# Supplementary material for: Development and application of a novel model to predict the risk of non-alcoholic fatty liver disease among lean pre-diabetics with normal blood lipid levels
Source: Lipids Health Dis. 2022 Dec 31;21:149. doi: 10.1186/s12944-022-01752-5 (PMC9804963; doi:10.1186/s12944-022-01752-5)
Supplement: Supplementary file 1 — Additional file 1: Table S1. Baseline characteristics of the participants in the cross-sectional Study 1 (N = 37,581). Table S2. Characteristics of the 3699 pre-diabetic individuals from Study 1, divided into training and validation sets. Table S3. Characteristics of the 7897 individuals enrolled in longitudinal Study 2, and 546 patients in the internal validation set. Table S4. Characteristics of the 4908 patients enrolled in external longitudinal cohort study 3, and 501 patients enrolled in the external validation set. [file 12944_2022_1752_MOESM1_ESM.docx]

# Supplementary Material

for

# Development and application of a novel model to predict the risk of non-alcoholic fatty liver disease among lean pre-diabetics with normal blood lipid levels

Wentao Zhu^1^, Pei Shi^1^, Jiwei Fu^1^, An Liang^1^, Ting Zheng^1^, Xiaoping Wu^1^*, Songsong Yuan^1^*

^1^Department of Infectious Diseases, the First Affiliated Hospital of Nanchang University, Nanchang, China

***Correspondance**

Xiaoping Wu, Department of Infectious Diseases, the First Affiliated Hospital of Nanchang University, No. 17 Yongwai Street, Donghu District, Nanchang, China

Email: [wuxiaoping2823@aliyun.co](mailto:wuxiaoping2823@aliyun.co)m

Songsong Yuan, Department of Infectious Diseases, the First Affiliated Hospital of Nanchang University, No. 17 Yongwai Street, Donghu District, Nanchang, China

Email: Yuansong52555@163.com

**Supplementary Tables**

**Table S1** Baseline characteristics of the participants in the cross-sectional Study 1 (N=37581)

| Characteristics | Cohorts in the cross-sectional study | | | *P* |
| --- | --- | --- | --- | --- |
|  | Normal | Pre-diabetes | Diabetes |  |
| Gender, Male/Female (n) | 13227/19982 | 2275/1424 | 551/122 | <0.001 |
| Age (years) | 35 (29-45) | 51 (39-68) | 64 (53-74) | <0.001 |
| NAFLD (n, %) | 977 (2.9%) | 287 (7.8%) | 100 (14.9%) | <0.001 |
| GGT (U/L) | 17 (13.0-22.0) | 20.0 (15.0-29.0) | 24.0 (18.0-35.0) | <0.001 |
| ALT (U/L) | 14.0 (11.0-19.0) | 16.0 (12.0-22.0) | 18.0 (13.0-23.0) | <0.001 |
| AST (U/L) | 19.0 (17.0-23.0) | 21.0 (18.0-25.0) | 21.0 (18.0-26.0) | <0.001 |
| ALT/AST (AAR) | 0.714 (0.600-0.875) | 0.750 (0.625-0.929) | 0.826 (0.682-1.000) | <0.001 |
| TP (g/L) | 73.2 (70.4-76.1) | 74.0 (71.0-77.0) | 73.3 (70.6-76.9) | <0.001 |
| ALB (g/L) | 44.6 (42.7-46.4) | 44.4 (42.5-46.3) | 43.9 (42.3-45.6) | <0.001 |
| GLB (g/L) | 28.7 (26.2-31.3) | 29.3 (26.7-32.1) | 29.4 (26.8-32.2) | <0.001 |
| TB (μmol/L) | 11.5 (9.1-14.8) | 11.8 (9.3-15.0) | 12.4 (9.8-15.3) | <0.001 |
| DBIL (μmol/L) | 1.8 (1.4-2.4) | 1.8 (1.4-2.4) | 2.0 (1.6-2.7) | <0.001 |
| BUN (mmol/L) | 4.05 (3.37-4.83) | 4.59 (3.76-5.54) | 5.20 (4.29-6.20) | <0.001 |
| Cr (μmol/L) | 73.0 (64.4-88.0） | 83.0 (69.6-95.0) | 84.0 (74.0-96.0) | <0.001 |
| UA (mmol/L) | 246.0 (200.0-307.0) | 274.0 (219.0-331.0) | 269.0 (227.0-323.5) | <0.001 |
| TC (mmol/L) | 4.27 (3.84-4.67) | 4.41 (3.95-4.79) | 4.44 (4.01-4.81) | <0.001 |
| TG (mmol/L) | 0.87 (0.68-1.11) | 0.99 (0.77-1.25) | 1.06 (0.81-1.31) | <0.001 |
| HDL-c (mmol/L) | 1.48 (1.30-1.69) | 1.44 (1.26-1.65) | 1.42 (1.25-1.63) | <0.001 |
| TG/HDL-c (THR) | 0.580 (0.429-0.797) | 0.682 (0.500-0.923) | 0.718 (0.536-1.007) | <0.001 |
| LDL-c (mmol/L) | 2.13 (1.82-2.43) | 2.24 (1.88-2.53) | 2.23 (1.90-2.52) | <0.001 |
| Height (m) | 1.63 (1.58-1.69) | 1.65 (1.59-1.70) | 1.65 (1.60-1.70) | <0.001 |
| Weight (Kg) | 54.0 (49.0-60.0) | 57.0 (52.0-62.0) | 58.0 (54.0-62.0) | <0.001 |
| BMI (kg/m^2^) | 20.44 (19.14-21.64) | 21.24 (19.95-22.20) | 21.67 (20.49-22.38) | <0.001 |

Abbreviations: AAR, ALT to AST ratio; ALB, albumin; ALT, alanine aminotransferase; AST, aspartate aminotransferase; BMI, body mass index; BUN, blood urea nitrogen; Cr, creatinine; DBIL, direct bilirubin; GGT, γ-glutamyltranspeptidase; GLB, globulin; HDL-C, high-density lipoprotein cholesterol; LDL-C, low-density lipoprotein cholesterol; NAFLD, nonalcoholic fatty liver disease; TB, total bilirubin; TC, total cholesterol; TG, triglyceride; THR, TG to HDL-C ratio; TP, total protein; UA, uric acid.

**Table S2** Characteristics of the 3699 pre-diabetic individuals from Study 1, divided into training and validation sets

| Characteristics | Total cohort (n=3699) | Training set (n=2774) | Validation set (n=925) | *P* |
| --- | --- | --- | --- | --- |
| Gender, male/female (n) | 2275/1424 | 1723/1051 | 552/373 | 0.419 |
| Age (years) | 51 (39-68) | 51 (39-68) | 53 (40-69) | 0.996 |
| NAFLD (n, %) | 287 (7.8%) | 227 (8.2%) | 60 (6.5%) | 0.248 |
| GGT (U/L) | 20 (15.0-29.0) | 20 (15.0-29.0) | 21.0 (16.0-29.0) | 0.998 |
| ALT (U/L) | 16.0 (12.0-22.0) | 16.0 (12.0-21.0) | 16.0 (12.0-22.0) | 0.244 |
| AST (U/L) | 21.0 (18.0-25.0) | 21.0 (18.0-25.0) | 21.0 (18.0-25.0) | 0.792 |
| AAR | 0.750 (0.625-0.929) | 0.741 (0.619-0.929) | 0.687 (0.503-0.916) | 0.542 |
| TP (g/L) | 74.0 (71.0-77.0) | 74.0 (71.0-77.0) | 73.9 (70.8-76.7) | 0.405 |
| ALB (g/L) | 44.4 (42.5-46.3) | 44.5 (42.6-46.4) | 44.3 (42.4-46.2) | 0.171 |
| GLB(g/L) | 29.3 (26.7-32.1) | 29.3 (26.8-32.1) | 29.1 (26.7-32.1) | 0.965 |
| TB (μmol/L) | 11.8 (9.3-15.0) | 11.8 (9.2-15.0) | 11.8 (9.4-14.8) | 0.777 |
| DBIL (μmol/L) | 1.8 (1.4-2.4) | 1.8 (1.4-2.4) | 1.7 (1.3-2.4) | 0.950 |
| BUN (mmol/L) | 4.59 (3.76-5.54) | 4.56 (3.72-5.50) | 4.60 (3.80-5.60) | 0.472 |
| Cr (μmol/L) | 83.0 (69.6-95.0) | 82.4 (69.0-95.0) | 83.6 (71.0-95.0) | 0.478 |
| UA (mmol/L) | 274.0 (219.0-331.0) | 272.5 (217.0-330.0) | 227.5 (226.0-333.0) | 0.988 |
| FPG (mmol/L) | 5.84 (5.69-6.10) | 5.83 (5.69-5.83) | 5.84 (5.69-6.10) | 0.829 |
| TC (mmol/L) | 4.41 (3.95-4.79) | 4.42 (3.97-4.80) | 4.39 (3.92-4.77) | 0.995 |
| TG (mmol/L) | 0.99 (0.77-1.25) | 1.00 (0.77-1.25) | 0.99 (0.78-1.24) | 0.910 |
| HDL-c (mmol/L) | 1.44 (1.26-1.65) | 1.45 (1.27-1.66) | 1.40 (1.23-1.63) | 0.781 |
| THR | 0.683 (0.500-0.924) | 0.681 (0.499-0.925) | 0.687 (0.503-0.916) | 1.000 |
| LDL-c (mmol/L) | 2.24 (1.89-2.53) | 2.24 (1.87-2.54) | 2.24 (1.89-2.52) | 0.969 |
| Height (m) | 1.65 (1.59-1.70) | 1.64 (1.59-1.70) | 1.65 (1.60-1.70) | 0.581 |
| Weight (kg) | 57.0 (52.0-62.0) | 57.0 (52.0-62.0) | 57.0 (52.0-62.0) | 0.867 |
| BMI (kg/m^2^) | 21.23 (19.95-22.20) | 21.22 (19.90-22.21) | 21.29 (20.00-22.19) | 0.901 |

Abbreviations: AAR, ALT to AST ratio; ALB, albumin; ALT, alanine aminotransferase; AST, aspartate aminotransferase; BMI, body mass index; BUN, blood urea nitrogen; Cr, creatinine; DBIL, direct bilirubin; FPG, fasting plasma glucose; GGT, γ-glutamyltranspeptidase; GLB, globulin; HDL-C, high-density lipoprotein cholesterol; LDL-C, low-density lipoprotein cholesterol; NAFLD, nonalcoholic fatty liver disease; TB, total bilirubin; TC, total cholesterol; TG, triglyceride; THR, TG to HDL-C ratio; TP, total protein; UA, uric acid

**Table S3** Characteristics of the 7897 individuals enrolled in longitudinal Study 2, and 546 patients in the internal validation set

|  | Internal longitudinal total cohort | | | | Internal validation set |
| --- | --- | --- | --- | --- | --- |
|  | Normal | Pre-diabetes | Diabetes | *P* |  |
| Gender, Male/Female (n) | 3549/3608 | 346/296 | 54/44 | 0.067 | 306/240 |
| Age (Years) | 38 (31-49) | 45 (33-59) | 48 (36-66) | <0.001 | 46 (33-61) |
| NAFLD (n, %) | 266 (3.7%) | 62 (9.7%) | 15 (15.3%) | <0.001 | 57 (10.4%) |
| ALP (U/L) | 65.0 (54.0-78.0) | 71.0 (58.0-88.0) | 75.5 (64.0-89.8) | <0.001 | 71.0 (58.0-88.0) |
| GGT (U/L) | 18.0 (15.0-24.0) | 22.0 (17.0-31.0) | 24.0 (18.0-39.0) | <0.001 | 22.0 (17.0-31.0) |
| ALT (U/L) | 14.0 (11.0-19.0) | 18.0 (14.0-24.0) | 20.0 (15.0-28.0) | <0.001 | 17.0 (12.8-23.0) |
| AST (U/L) | 20.0 (17.0-23.0) | 22.0 (19.0-26.0) | 23.0 (19.0-28.0) | <0.001 | 22.0 (19.0-26.0) |
| AAR | 0.714 (0.600-0.882) | 0.750 (0.609-0.930) | 0.852 (0.682-1.060) | <0.001 | 0.750 (0.609-0.930) |
| TP (g/L) | 73.7 (71.0-76.4) | 74.0 (71.2-76.9) | 74.0 (71.3-77.0) | 0.055 | 74.0 (71.0-76.8) |
| ALB (g/L) | 44.4 (42.6-46.1) | 44.6 (42.7-46.4) | 43.8 (41.8-45.9) | 0.024 | 44.6 (42.7-46.4) |
| GLB (g/L) | 29.2 (26.9-31.7) | 29.3 (27.0-32.2) | 30.2 (27.7-34.3) | 0.012 | 29.3 (26.9-30.0) |
| TB (μmol/L) | 11.1 (8.8-14.3) | 11.2 (9.1-14.3) | 12.0 (9.3-14.9) | 0.125 | 11.4 (9.0-14.4) |
| DBIL (μmol/L) | 2.1 (1.5-2.8) | 2.1 (1.6-3.1) | 2.4 (1.9-3.1) | 0.005 | 2.1 (1.5-3.0) |
| BUN (mmol/L) | 4.20 (3.50-5.00) | 4.90 (3.90-5.84) | 5.50 (4.32-6.82) | <0.001 | 4.99 (3.97-5.90) |
| Cr (μmol/L) | 71.0 (62.0-84.0) | 81.0 (69.0-95.0) | 83.0 (72.0-95.5) | <0.001 | 85.0 (72.0-98.0) |
| UA (mmol/L) | 240.0 (198.0-299.0) | 268.0 (223.0-330.3) | 257.5 (231.3-322.5) | <0.001 | 284.0 (230.0-339.0) |
| FPG (mmol/L) | 4.89 (4.67-5.14) | 5.85 (5.70-6.11) | 7.69 (7.16-9.25) | <0.001 | 5.86 (5.70-6.10) |
| TC (mmol/L) | 4.36 (3.95-4.74) | 4.45 (4.02-4.84) | 4.30 (3.92-4.82) | <0.001 | 4.44 (3.99-4.82) |
| TG (mmol/L) | 0.88 (0.70-1.13) | 1.01 (0.79-1.23) | 1.04 (0.77-1.34) | <0.001 | 1.02 (0.80-1.24) |
| HDL-c (mmol/L) | 1.51 (1.30-1.74) | 1.47 (1.27-1.69) | 1.40 (1.24-1.59) | <0.001 | 1.46 (1.27-1.69) |
| THR | 0.58 (0.43-0.80) | 0.69 (0.52-0.89) | 0.70 (0.51-1.07) | <0.001 | 0.692 (0.526-0.892) |
| LDL-c (mmol/L) | 2.09 (1.81-2.37) | 2.18 (1.87-2.45) | 2.16 (1.84-2.46) | <0.001 | 2.18 (1.86-2.46) |
| Height (m) | 1.63 (1.58-1.68) | 1.64 (1.60-1.70) | 1.65 (1.60-1.69) | 0.001 | 1.65 (1.60-1.70) |
| Weight (kg) | 53.7 (49.4-58.8) | 56.6 (51.4-61.0) | 60.0 (52.8-61.4) | <0.001 | 57.7 (52.1-61.9) |
| BMI (kg/m^2^) | 20.37 (19.17-21.54) | 21.03 (19.90-22.12) | 21.29 (20.16-22.21) | <0.001 | 21.13 (19.96-22.19) |
| SBP (mmHg) | 113.0 (105.0-122.0) | 128.0 (117.0-140.0) | 132.0 (120.8-148.5) | <0.001 | 130.0 (119.0-140.0) |
| DBP (mmHg) | 68.0 (63.0-75.0) | 74.0 (68.0-82.0) | 75.0 (68.0-82.0) | <0.001 | 75.0 (68.0-82.5) |
| Follow-up (months) | 36 (24-47) | 35 (23-47) | 36 (23-48) | <0.001 | 25 (22-39) |

Abbreviations: AAR, ALT to AST ratio; ALB, albumin; ALP, alkaline phosphatase; ALT, alanine aminotransferase; AST, aspartate aminotransferase; BMI, body mass index; BUN, blood urea nitrogen; Cr, creatinine; DBIL, direct bilirubin; DBP, diastolic blood pressure, FPG, fasting plasma glucose; GGT, γ-glutamyltranspeptidase; GLB, globulin; HDL-C, high-density lipoprotein cholesterol; LDL-C, low-density lipoprotein cholesterol; NAFLD, nonalcoholic fatty liver disease; SBP, systolic blood pressure, TB, total bilirubin; TC, total cholesterol; TG, triglyceride; THR, TG to HDL-C ratio; TP, total protein; UA, uric acid.

**Table S4** Characteristics of the 4908 patients enrolled in external longitudinal cohort study 3, and 501 patients enrolled in the external validation set

| Characteristics | External longitudinal cohort | |  | External validation set |
| --- | --- | --- | --- | --- |
|  | Non-NAFLD | NAFLD | *P* |  |
| Gender, Male/Female (n) | 1698/3067 | 110/33 | <0.001 | 308/193 |
| Age (Years) | 39 (35-45) | 43 (39-50) | <0.001 | 42 (37-50) |
| NAFLD (n,%) |  |  |  | 39 (7.8%) |
| GGT (U/L) | 12.0 (10.0-15.0) | 16.0 (13.0-23.0) | <0.001 | 14.0 (11.0-19.0) |
| ALT (U/L) | 14.0 (11.0-17.0) | 21.0 (16.0-29.0) | <0.001 | 16.0 (12.0-20.0) |
| AST (U/L) | 16.0 (13.0-19.0) | 18.0 (15.0-23.0) | <0.001 | 17.0 (13.0-20.0) |
| AAR | 0.889 (0.733-1.087) | 1.188 (0.941-1.457) | <0.001 | 1.00 (0.78-1.21) |
| FPG (mmol/L) | 5.00 (4.72-5.22) | 5.27 (5.05-5.55) | <0.001 | 5.66 (5.55-5.83) |
| HbA1c (%) | 5.10 (4.90-5.35) | 5.20 (5.00-5.50) | <0.001 | 5.50 (5.17-5.70) |
| TC (mmol/L) | 4.55 (4.19-4.86) | 4.73 (4.34-4.99) | 0.001 | 4.63 (4.24-4.94) |
| TG (mmol/L) | 0.51 (0.37-0.71) | 0.81 (0.56-1.04) | <0.001 | 0.60 (0.44-0.84) |
| HDL-c (mmol/L) | 1.53 (1.32-1.76) | 1.28 (1.14-1.44) | <0.001 | 1.42 (1.22-1.68) |
| THR | 0.332 (0.223-0.496) | 0.613 (0.418-0.864) | <0.001 | 0.41 (0.27-0.64) |
| WC (cm) | 70.0 (66.0-74.6) | 77.0 (74.5-80.0) | <0.001 | 73.5 (69.0-77.2) |
| Weight (kg) | 52.8 (47.9-58.7) | 61.6 (55.4-66.0) | <0.001 | 57.1 (51.4-62.6) |
| BMI (kg/m^2^) | 20.05 (18.76-21.36) | 21.84 (20.88-22.58) | <0.001 | 20.82 (19.23-21.88) |
| SBP (mmHg) | 118.0 (100.5-124.0) | 109.5 (109.0-111.5) | 0.197 | 104.0 (97.0-120.5) |
| DBP (mmHg) | 66.0 (60.5-72.5) | 71.0 (65.5-77.0) | <0.001 | 70.0 (64.0-76.0) |
| Never smoked | 3466 (72.7%) | 76 (53.1) | <0.001 | 294 (58.7%) |
| Ex-smoker | 626 (13.1%) | 32 (22.4%) | <0.001 | 106 (21.2%) |
| Current smoker | 673 (14.1%) | 35 (24.5%) | <0.001 | 101 (20.2%) |
| Regular exerciser | 824 (17.3%) | 32 (22.4%) | 0.114 | 89 (17.8%) |
| Follow-up (Days) | 2037.0 (1046.5-3359.5) | 2147.0 (794.0-3642.0) | 0.830 | 1638 (734.5-2926.0) |

Abbreviations: AAR, ALT to AST ratio; ALT, alanine aminotransferase; AST, aspartate aminotransferase; BMI, body mass index; DBP, diastolic blood pressure; FPG, fasting plasma glucose; GGT, γ-glutamyltranspeptidase; HbA1c, hemoglobin A1c; HDL-C, high-density lipoprotein cholesterol; NAFLD, nonalcoholic fatty liver disease; SBP, systolic blood pressure; TC, total cholesterol; TG, triglyceride; THR, TG to HDL-C ratio; WC, waist circumferenc.
